# Factors affecting intention to screen after being informed of benefits and harms of breast cancer screening: a study in 5 European countries in 2021

**DOI:** 10.1186/s13690-022-00902-6

**Published:** 2022-05-23

**Authors:** David Ritchie, Guido Van Hal, Stephan Van den Broucke

**Affiliations:** 1 Faculty of Medicine and Health Sciences, Campus Drie Eiken, Universiteitsplein 1, 2610 Wilrijk, Belgium; 2 Faculty of Psychology and Educational Sciences, UC Louvain, Louvain-la-Neuve, Belgium

**Keywords:** Health literacy, Informed choice, Breast cancer, Mass screening

## Abstract

**Background:**

Participation in mammography screening comes with harms alongside benefits. Information about screening provided to women should convey this information yet concerns persist about its effect on participation. This study addressed factors that may influence the intention to screen once a woman has been informed about benefits and harms of participation.

**Methods:**

A cross-sectional survey of women from five countries (Belgium, France, Italy, Spain, and the United Kingdom) was performed in January 2021. The survey contained a statement regarding the benefits and harms of mammography screening along with items to measure cognitive variables from the theory of planned behaviour and health belief model and the 6-item version of the European Health Literacy Survey Questionnaire (HLS-EU-Q6). Logistic regression and mediation analysis were performed to investigate the effect of cognitive and sociodemographic variables.

**Results:**

A total of 1180 participants responded to the survey. 19.5% of participants (*n* = 230) were able to correctly identify that mammography screening carries both benefits and harms. 56.9% of participants (*n* = 672) responded that they would be more likely to participate in screening in the future after being informed about the benefits and harms of mammography screening. Perceived behavioural control and social norms demonstrated were significant in predicting intention, whereas, the effect of health literacy was limited.

**Conclusions:**

Informing women about the presence of benefits and harms of in mammography screening participation did not negatively impact upon intention to be screened. Information should also address perception on implementation factors alongside messages on benefits and harms. Overall, screening programme managers should not be discouraged by the assumption of decreased participation through increasing efforts to address the lack of knowledge on benefits and harms.

**Supplementary Information:**

The online version contains supplementary material available at 10.1186/s13690-022-00902-6.

## Background

Breast cancer is the most commonly diagnosed cancer, with an estimated 1.1 million newly diagnosed cases annually on a global scale [1]. Due to the relatively favourable prognosis breast cancer is also the most prevalent cancer worldwide, yet it remains the most common cancer-related cause of death in women globally [2]. Considering the significant burden of breast cancer to public health, the World Health Organization (WHO) has launched the Global Breast Cancer Initiative (GBCI) to reduce breast cancer deaths by 2.5% per year between 2020 and 2040 [3]. A key pillar of this initiative is to promote early detection of the cancer. For high-income countries, organised quality assured mammographic screening remains a strongly recommended tool [4].

The widespread use of breast cancer screening using mammography over the past decades has been associated with a steep decline in breast cancer mortality during that time [5, 6]. However, the effectiveness of mammography screening in terms of its contribution to reducing mortality has been called into question in recent times and is increasingly becoming the subject of debate [7, 8]. Consequently, the imperative for screening programmes and health professionals to facilitate an informed choice about mammography screening amongst eligible women has been explicitly recommended in the latest update of the European guidelines on quality assurance in breast cancer screening [9].

Informed choice involves a conscious decision that is made based on relevant knowledge, is consistent with the individual’s personal values, and is subsequently acted upon [10]. In the context of breast cancer screening, informed choice comprises participation or intention to participate in breast cancer screening following a presentation of the benefits and harms of participation [11]. Thus, to make an informed choice about their participation in breast cancer screening, women need to be informed about both the benefits and the harms of mammography screening. Whilst this principle has been acknowledged as an intrinsic ethical requirement for a quality assured breast screening programme [12], the difficulty of estimating harms such as overdiagnosis [13], and the lack of consensus on the appropriate measures and tools to provide this information to women [14], continue to present obstacles for delivering informed choice in practice [15]. Against this background, studies suggest that women in European countries, which have widespread and established breast cancer screening programmes, have limited knowledge about the benefits and harms of breast screening, which impedes their ability to make a truly informed choice [16, 17].

This lack of accurate knowledge is closely linked to the issue of health literacy. Health literacy entails people’s knowledge, motivation, and competences to access, understand, appraise, and apply health information to make judgments and take decisions in everyday life concerning healthcare, disease prevention and health promotion to maintain or improve quality of life during the life course [18]. It has been associated with the uptake of prevention and early detection services such as breast cancer screening, with low health literate people being less likely to participate [19]. Low participation in services such as breast screening has also been frequently reported amongst people with low socio-economic status [20, 21]. As health literacy can be considered to act as a mediator between socio-economic status and health inequalities [22, 23], addressing health literacy specific to the benefits and harms of breast cancer screening may prove a worthwhile strategy to facilitate informed choice among women, which may potentially improve equity in participation.

Against the backdrop of breast screening programmes in Europe seeking to promote informed choice, this study aimed to investigate if informing women that participation in breast screening carries with it both benefits and harms affects their intention to be screened. The study also sought to assess the influence that cognitive variables from behavioural theories, health literacy and sociodemographic factors may have on intention.

## Methods

A cross-sectional survey of women aged 50 years and above from five countries (Belgium, France, Italy, Spain, and the United Kingdom [UK]) was performed in January 2021. The sample size for the total number of respondents was guided by first determining the sum of the estimated population of women aged 50 years old and above in the five countries in 2020. Applying a 95% confidence level and 3% margin of error, a minimum total sample of 1068 was computed, which would entail a minimum of 214 per country.

**Fig. 1:**
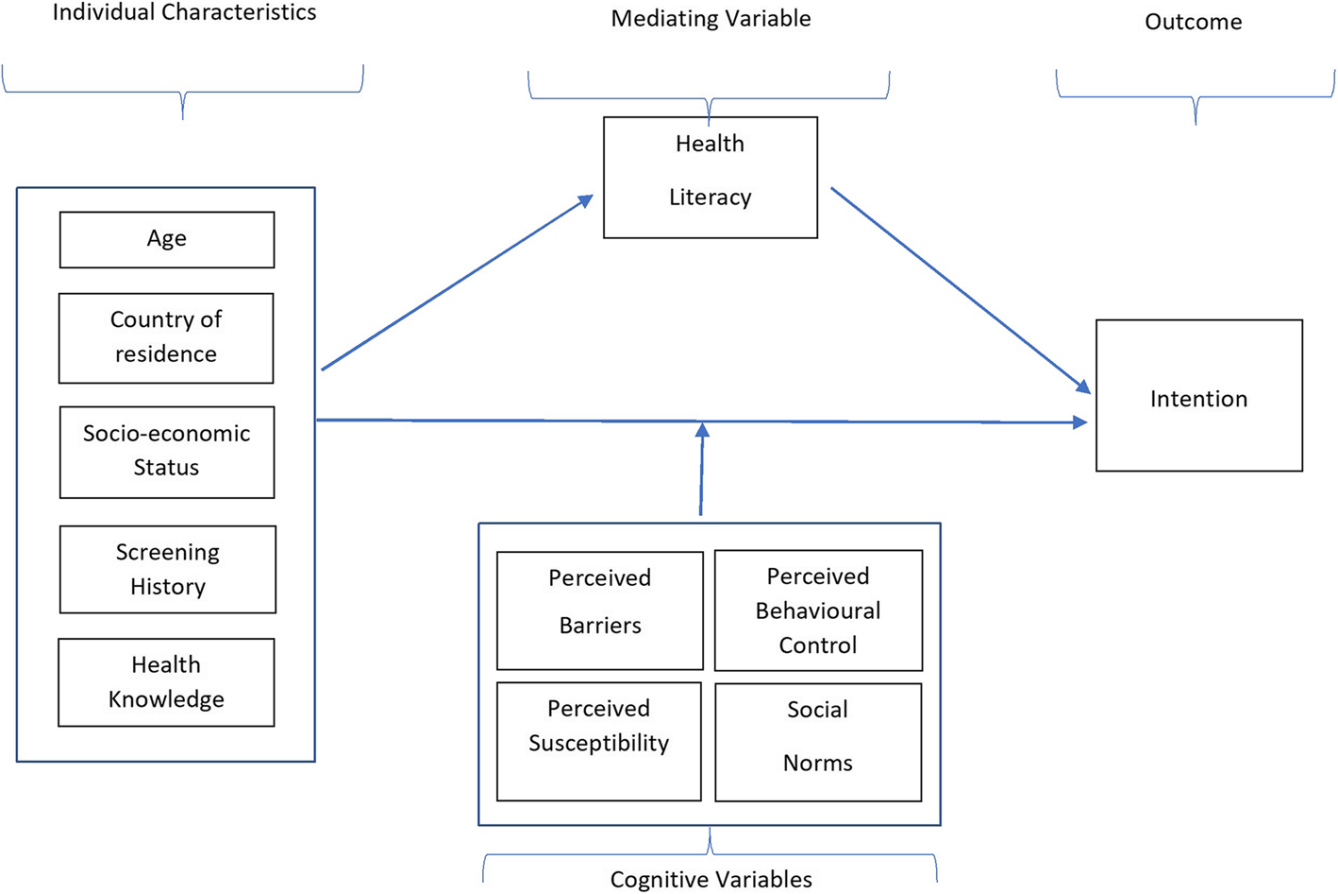
Conceptual model for development of items for the survey on mammography screening targeting women in 5 European countries, 2021

An online survey was developed in English and translated into the national languages of the five countries surveyed (Annex 1). To inform the questionnaire items a conceptual model was developed based on the Theory of Planned Behaviour (TPB) and the Health Belief Model (HBM) (Fig. 1). The model includes individual characteristics of participants, several cognitive variables informed by the models of behaviour.

Individual characteristics were concerned with age (reported in dichotomous categories of age 50–59 years old, and age 60 years old and above), country of residence (Belgium, France, Italy, Spain, and the UK), and level of household income per annum (< 19,000€; 20,000-39,999€; 40,000-59,999€; 60,000-79,999€; > 80,000€) of participants. For the UK, the household income reported in Pound Sterling (GBP) was converted to Euros. The respondents’ history of participation in breast cancer screening. Participants were also asked if they had ever participated in mammography screening, with an option to define if they had been invited by the mammography screening programme or referred by a health professional. This was later dichotomized for the analysis as participated or not participated. Knowledge of the benefits and harms of breast cancer screening was assessed by asking participants whether they could currently identify that mammography came with benefits and risks, or if they believed it came with benefits but no risks, or its goal was to prevent cancer before it occurs. The outcome was dichotomised to either correctly identifying breast cancer screening has benefit and harms or not. Intention to be screened was operationalised by asking participants, after being presented with the correct statement that breast cancer screening carries both benefits and harms, whether this made them more likely to participate in screening, less likely to participate, or neither more nor less likely.

Cognitive variables measured by the survey were perceived social norms, perceived behavioural control, perceived susceptibility, and perceived barriers to screening. The items were informed by the Champion Health Belief Model Scale (CHBMS) used previously in studies on attitudes to breast cancer screening [24]. Perceived social norms was measured by asking participants whether they believed that ‘most people who are important to me think I should have my breasts screened’; perceived susceptibility by asking participants whether they believed that ‘my chances of getting breast cancer in the next few years are great’; and perceived barriers by asking them whether they believed that ‘I have other problems more important than getting a mammogram’. For all three these items, a 4-point Likert scale was used with options ‘strongly disagree’, ‘disagree’, ‘agree’, and ‘strongly agree’, or to answer, ‘I am not sure’. Perceived behavioural control was measured by asking participants whether they believed that ‘Keeping my appointment for breast cancer screening will be … ‘, using a 4-point Likert scale ranging from very easy to easy, difficult, or very difficult, with an option to answer, ‘I am not sure’. For each variable, items were dichotomized in the subsequent analysis to the categories of ‘agree/disagree’ or ‘easy/difficult’.

Health literacy was measured using the 6-item version of the European Health Literacy Survey Questionnaire (HLS-EU-Q6) [25], using a 4-point Likert scale per item (very difficult, difficult, easy, very easy). Answers were coded on a scale from 1 to 4 (‘very difficult’ scoring 1; ‘very easy’ scoring 4). The Health Literacy score is then calculated as a mean of the scores of the completed items in the HLS-EU-Q6 Questionnaire (sum of answers/number of items). This presents a mean score that can range from 1 to 4. Three levels for the scale have been defined and validated against the more extensive 47 item version of the European Health Literacy Survey Questionnaire: Inadequate Health Literacy (≤2); Limited Health Literacy (> 2 and ≤ 3); Sufficient Health Literacy (> 3) [22]. Cronbach’s alpha for the HLS-EU-Q6 score was calculated to check internal consistency on the data of each participating country separately.

A convenience sample of ten women pre-tested the survey for intelligibility prior to translation, which revealed no problems in the construction of the questionnaire.

The questionnaire was transposed to a web-based survey platform administered by Panelbase UK, which is a research consultancy that delivers online surveys to an established panel drawn from the general population who have provided consent to be included in such research. Panelbase distributed the survey to active users in its panel (defined as having completed one survey in the past twelve months) meeting the eligibility criteria of age and country of residence. Eligible potential participants were contacted via email with a link to the survey. This process ensures that only the people contacted are allowed to participate. Only this sample of panel members has access to the survey via their username and password, and respondents to the survey can only ever answer the survey once to avoid duplicate results. The survey remained open until the minimal sample size was exceeded. Incomplete responses or responses with missing values were excluded. Due to the lag in validating responses, greater eligible responses per country were reported than the minimum sample size requested.

Descriptive statistics were used to present absolute and relative frequencies of the dichotomised variables. Correlation analyses were performed to inspect the association between intention to screen and the antecedent variables included in the conceptual model.

Logistic regression analysis was applied to test two models explaining the intention to be screened for breast cancer: a first model testing the influence of cognitive variables, plus screening history and health knowledge on screening intention, and a second model adjusting the first model for age and household income per annum. Adjusted odds ratios (ORs) are reported with 95% confidence intervals (CIs), with significance set at *p* < 0.05. A mediation analysis using the Baron andKenney method [26] and bootstrapping was performed to examine the influence of health literacy (measured via HLS-EU-Q6 mean score) on the relationship between age, household income (as a proxy of socio-economic status), screening history, and health knowledge (independent variables) and intention to screen (dependent variable). PROCESS v3.5 using the Hayes method was calculated for the multi-categorical variable of household income per annum. Data were analysed using IBM SPSS Statistics for Windows, V.27.0 (IBM).

## Results

A total of 1180 participants responded to the survey from five countries (Belgium, France, Italy, Spain, and the UK). Participants per country ranged from 228 (19.3% of total sample) to 239 (20.3% of total sample). Age of participants was reported only in the categories of aged 50–59 years old and 60 years and older. For the total sample, 55.1% were aged 50–59 years old and 44.9% were aged 60 years and older. As a proxy of socio-economic status, household income per annum was asked to participants. A total of 947 participants provided a response. The most frequent range of household income per annum for the total sample was €20,000 - €39,999, which was reported by 388 participants (32.9% of the total sample). The least frequent option for household income per annum for the total sample was €80,000+, which was reported by 29 participants (2.5% of the total sample). Two hundred thirty-three participants (19.7% of sample) declined to provide data on household income per annum. The characteristics of the participants are represented in Table 1.

**Table 1:** Characteristics of women in 5 European countries responding to the survey on mammography screening, 2021 (*n* = 1180)

		Country											
		Belgium		France		Italy		Spain		UK		Total	
		N	%	N	%	N	%	N	%	N	%	N	%
Country	Belgium	236	100.0	0	0.0	0	0.0	0	0.0	0	0.0	236	20.0
	France	0	0.0	238	100.0	0	0.0	0	0.0	0	0.0	238	20.2
	Italy	0	0.0	0	0.0	239	100.0	0	0.0	0	0.0	239	20.3
	Spain	0	0.0	0	0.0	0	0.0	228	100.0	0	0.0	228	19.3
	UK	0	0.0	0	0.0	0	0.0	0	0.0	239	100.0	239	20.3
	Total	236	100.0	238	100.0	239	100.0	228	100.0	239	100.0	1180	100.0
Age Range	50–59 y/o	83	35.2	127	53.4	148	61.9	172	75.4	120	50.2	650	55.1
	60+ y/o	153	64.8	111	46.6	91	38.1	56	24.6	119	49.8	530	44.9
	Total	236	100.0	238	100.0	239	100.0	228	100.0	239	100.0	1180	100.0
Household Income per Annum	Prefer not to say	85	36.0	43	18.1	44	18.4	35	15.4	26	10.9	233	19.7
	<€19,999	50	21.2	73	30.7	56	23.4	69	30.3	76	31.8	324	27.5
	€20,000–€39,999	59	25.0	76	31.9	87	36.4	78	34.2	88	36.8	388	32.9
	€40,000–€59,999	21	8.9	38	16.0	31	13.0	33	14.5	31	13.0	154	13.1
	€60,000–€79,999	11	4.7	6	2.5	14	5.9	8	3.5	13	5.4	52	4.4
	€80,000+	10	4.2	2	0.8	7	2.9	5	2.2	5	2.1	29	2.5
	Total	236	100.0	238	100.0	239	100.0	228	100.0	239	100.0	1180	100.0

Participants were asked about their history of participation in breast cancer screening. In total, 90.3% of survey participants had participated at least one-time in breast cancer screening, with the percentages per country ranging from 89.9% (France) to 91.2% (Spain). Only 19.5% of participants (*n* = 230) were able to correctly identify that breast cancer screening carries both benefits and harms, with country percentages being the lowest in Italy (13.4%) and the highest in the UKL (25.4%). When asked to rate their intention to participate in breast cancer screening after being informed that screening for breast cancer involves harms such as overdiagnosis as well as benefits for the participating woman, just 4.8% (*n* = 57) of the total sample stated they would be *less* likely to participate after being presented with the information on benefits and harms of breast cancer screening. This percentage ranged from 4.2% (Italy) to 5.5% (France). Of the remaining participants, 38.2% (*n* = 451) reported that they would be *neither more nor less likely* to participate in the future as a result of being informed of the information on benefits and harms (with country percentages ranging from 5.3% for Spain to 55.2% for the UK), whereas 56.9% of participants (*n* = 672) responded that they would be *more* likely to participate in breast cancer screening in the future after being informed about the benefits and harms of breast cancer screening. For the latter, country percentages ranged from 40.2% (UK) to 89.9% (Spain).

The mean score on the HLS-EU-Q6 for all participants in all countries was 2.56 (Standard deviation [SD] = .48). The HLS-EU-Q6 mean scores for each country followed a normal distribution, with a median of 2.5 and the mode of 2.67. By country the mean score of the HLS-EU-Q6 was Belgium 2.53 (SD = .41); France 2.57 (SD = .52); Italy 2.52 (SD = .47); Spain 2.54 (SD = .51); United Kingdom 2.67 (SD = .48).

For all countries combined, most participants had a mean score in the range of the category ‘limited health literacy’ (*n* = 853; 72.3%). Fewer participants were categorised as possessing ‘sufficient health literacy’ (*n* = 252; 21.4%), whilst the fewest participants from all countries categorised had a mean score in the range of the category ‘inadequate health literacy’ (*n* = 75; 6.4%). The categories of health literacy were mostly consistent across countries: the proportion of participants categorised as possessing ‘inadequate health ranged from 3.3% (United Kingdom) to 8% (France); for participants categorised as possessing ‘limited health literacy’ the proportion ranged from 68.5% (France) to 75.8% (Belgium); and for participants categorised as possessing ‘sufficient health literacy’ the proportion ranged from 17.4% (Belgium) to 26.8 (United Kingdom).

Regarding social norms, many participants (*n* = 755, 64% of the total sample) reported that people close to them believe they should be screened for breast cancer. This result varied from 57.6% (Belgium) to 69.1% (France) by country. Most participants (*n* = 921, 78.1% of the total sample) thought that it would be easy to keep an appointment for breast screening (ranging from 72% for Italy to 83.3% for the UK). From the total sample, 60.7% (*n* = 716) did not know whether their likelihood of getting breast cancer in next few years was great or not, with the response by country ranging from 52.5% (France) to 72.4% (Spain). Two thirds (66%) of the participants (*n* = 779) reported that they have no other problems more important than getting a mammogram, with scores per country varying from 59.3% (Belgium) to 71.1% (UK). The frequencies and descriptive statistics of the survey responses are shown in Table 2.

**Table 2:** Relative frequencies of the responses by women in 5 European countries responding to the survey on mammography screening, 2021 (n = 1180)

		Country											
		Belgium		France		Italy		Spain		UK		Total	
		N	%	N	%	N	%	N	%	N	%	N	%
**Intention** *Intention to participate after being informed about benefits and harms*	More likely to participate	96	40.7	121	50.8	154	64.4	205	89.9	96	40.2	672	56.9
	Neither more nor less likely to participate	128	54.2	104	43.7	75	31.4	12	5.3	132	55.2	451	38.2
	Less likely to participate	12	5.1	13	5.5	10	4.2	11	4.8	11	4.6	57	4.8
**Screening History** *Has Participated in Breast Cancer Screening*	Yes	213	90.3	214	89.9	216	90.4	208	91.2	215	90.0	1066	90.3
	No	23	9.7	24	10.1	23	9.6	20	8.8	24	10.0	114	9.7
**Health Knowledge** *Aware that Breast Cancer Screening has Benefits and Harms*	Correct	56	23.7	49	20.6	32	13.4	31	13.6	62	25.9	230	19.5
	Incorrect	180	76.3	189	79.4	207	86.6	197	86.4	177	74.1	950	80.5
**Health Literacy** *HLS-EU-Q6 Score*	Inadequate Health Literacy (≤2)	16	6.8	19	8.0	16	6.7	16	7.0	8	3.3	75	6.4
	Limited Health Literacy (> 2 and ≤ 3)	179	75.8	163	68.5	177	74.1	167	73.2	167	69.9	853	72.3
	Sufficient Health Literacy (> 3)	41	17.4	56	23.5	46	19.2	45	19.7	64	26.8	252	21.4
**Social Norms** *Most people who are important to me think I should have my breasts screened*	I am not sure	44	18.6	50	21.0	60	25.1	51	22.4	56	23.4	261	22.1
	Disagree	29	12.3	51	21.4	40	16.7	22	9.6	22	9.2	164	13.9
	Agree	163	69.1	137	57.6	139	58.2	155	68.0	161	67.4	755	64.0
**Perceived Behavioural Control** *Keeping my appointment for breast cancer screening will be* …	I am not sure	24	10.2	22	9.2	54	22.6	41	18.0	23	9.6	164	13.9
	Difficult	19	8.1	33	13.9	13	5.4	13	5.7	17	7.1	95	8.1
	Easy	193	81.8	183	76.9	172	72.0	174	76.3	199	83.3	921	78.1
**Perceived Susceptibility** *My chances of getting breast cancer in the next few years are great*	I am not sure	139	58.9	125	52.5	131	54.8	165	72.4	156	65.3	716	60.7
	Disagree	74	31.4	93	39.1	92	38.5	20	8.8	54	22.6	333	28.2
	Agree	23	9.7	20	8.4	16	6.7	43	18.9	29	12.1	131	11.1
**Perceived Barriers** *I have other problems more important than getting a mammogram*	I am not sure	47	19.9	39	16.4	56	23.4	30	13.2	35	14.6	207	17.5
	Disagree	140	59.3	144	60.5	158	66.1	167	73.2	170	71.1	779	66.0
	Agree	49	20.8	55	23.1	25	10.5	31	13.6	34	14.2	194	16.4

Chi-square tests on the association between intention to screen in the future (after having been informed of benefits and risks of breast screening) with age, household income, screening history, knowledge of benefits and harms of breast cancer screening (health knowledge), health literacy, social norms, perceived behavioural control, perceived susceptibility and perceived barriers gave statistically significant associations between all variables with the exception of household income (*p* = .300) and health literacy (*p* = .401).

Table 3 shows the logistic regression analysis of two models to predict screening intention. Table 3 shows the variables included in the two models, reporting the strength of association with intention to screen for each element of the variables. This allows for simple comparison, for instance, older women are less likely (OR = 0.51) to indicate that they would be more likely to participate next time invited compared to women in the age group 50–59 y.

**Table 3:** Adjusted Odds Ratio and 95% Confidence Intervals (CI) for logistic regression models predicting intention to screen by women in 5 European countries responding to the survey on mammography screening, 2021 (n = 1180)

Components	Model 1^**a,b**^				Model 2^**c,d**^			
	Neither more nor less likely to participate next time invited/referred for breast cancer screening		More likely to participate next time invited/referred for breast cancer screening		Neither more nor less likely to participate next time invited/referred for breast cancer screening		More likely to participate next time invited/referred for breast cancer screening	
	Adjusted OR	95% CI	Adjusted OR	95% CI	Adjusted OR	95% CI	Adjusted OR	95% CI
**Age**								
50–59 y	.	.	.	.	.	.	.	.
60+ y	.	.	.	.	.837	(.458–1.531)	.505	(.274–.931)
**Income**								
€20,000–€39,999	.	.	.	.	.	.	.	.
< €19,999	.	.	.	.	1.446	(.663–3.151)	1.145	(.522–2.515)
€40,000–€59,999	.	.	.	.	1.169	(.456–2.997)	.788	(.303–2.044)
€60,000–€79,999	.	.	.	.	.742	(.206–2.673)	.419	(.114–1.538)
€80,000+	.	.	.	.	2.967	(.322–27.361)	2.585	(.276–24.239)
Prefer not to say	.	.	.	.	1.683	(.706–4.012)	1.154	(.479–2.782)
**HLS6 Score**	.911	(.769–1.078)	.847	(.714–1.004)	.903	(.759–1.073)	.843	(.708–1.004)
**Screening History**								
Not participated	.	.	.	.	.	.		.
Participated	.812	(.387–1.703)	1.780	(.803–3.947)	.778	(.366–1.657)	1.883	(.833–4.257)
**Health Knowledge**								
Incorrect	.	.	.	.	.	.	.	.
Correct	.622	(.302–1.284)	.991	(.472–2.078)	.618	(.298–1.283)	1.061	(.502–2.242)
**Social Norm**								
Agree	.	.	.	.	.	.	.	.
Disagree	.323	(.153–.686)	.345	(.162–.734)	.322	(.150–.691)	.351	(.163–.758)
Unsure	.754	(.364–1.560)	.452	(.216–.946)	.766	(.369–1.594)	.447	(.212–.940
**Perceived Behavioural Control**								
Easy	.	.	.	.	.	.	.	.
Difficult	.237	(.103–.544)	.140	(.059–.335)	.217	(.093–.504)	.120	(.049–.290)
Unsure	.201	(.094–.430)	.142	(.066–.305)	.194	(.090–.421)	.132	(.060–.288)
**Perceived Susceptibility**								
Unsure	.	.	.	.	.	.	.	.
Agree	.810	(.215–3.048)	1.877	(.511–6.889)	.864	(.225–3.316)	1.901	(.506–7.138)
Disagree	.729	(.390–1.364)	.457	(.242–.865)	.696	(.369–1.310)	.435	(.228–.830)
**Perceived Barriers**								
Disagree	.	.	.	.	.	.	.	.
Agree	.338	(.166–.692)	.175	(.084–.362)	.347	(.169–.711)	.183	(.088–.382)
Unsure	.638	(.280–1.454)	.508	(.222–1.161)	.643	(.279–1.481)	.544	(.235–1.259)

^a^ Model 1 result = Negelkerke R^2^ .201

^b^ Model 1 reference category: Less likely to participate next time invited/referred for breast cancer screening

^c^ Model 2 result = Negelkerke R^2^ .224

^d^ Model 2 reference category: Less likely to participate next time invited/referred for breast cancer screening

Model 1 was comprised of the following variables derived from the survey items: history of screening participation, knowledge of benefits and harms of breast cancer screening, level of health literacy, social norms, perceived behavioural control, perceived susceptibility, and perceived barriers on screening intention. This model was significant (χ2(22) = 210.553, *p* < .001) explaining 20.1% if the variance of screening intention (Nagelkerke R^2^ = .201) and correctly classifying 63.1% of cases. Of the variables included in the model, Perceived Behavioural Control demonstrated a strong significance in its effect on screening intention.

Model 2 built upon the variables included in Model 1 with the addition of the socio-demographic variables of age and household income. The addition of these variables improved marginally the explained variance (Nagelkerke R^2^ = .224; χ2(34) = 236.411, *p* < .001) but only classified 61.9% of the cases correctly. As with Model 1, Perceived Behavioural Control displayed clear significance in its effect on screening intention.

**Fig. 2:**
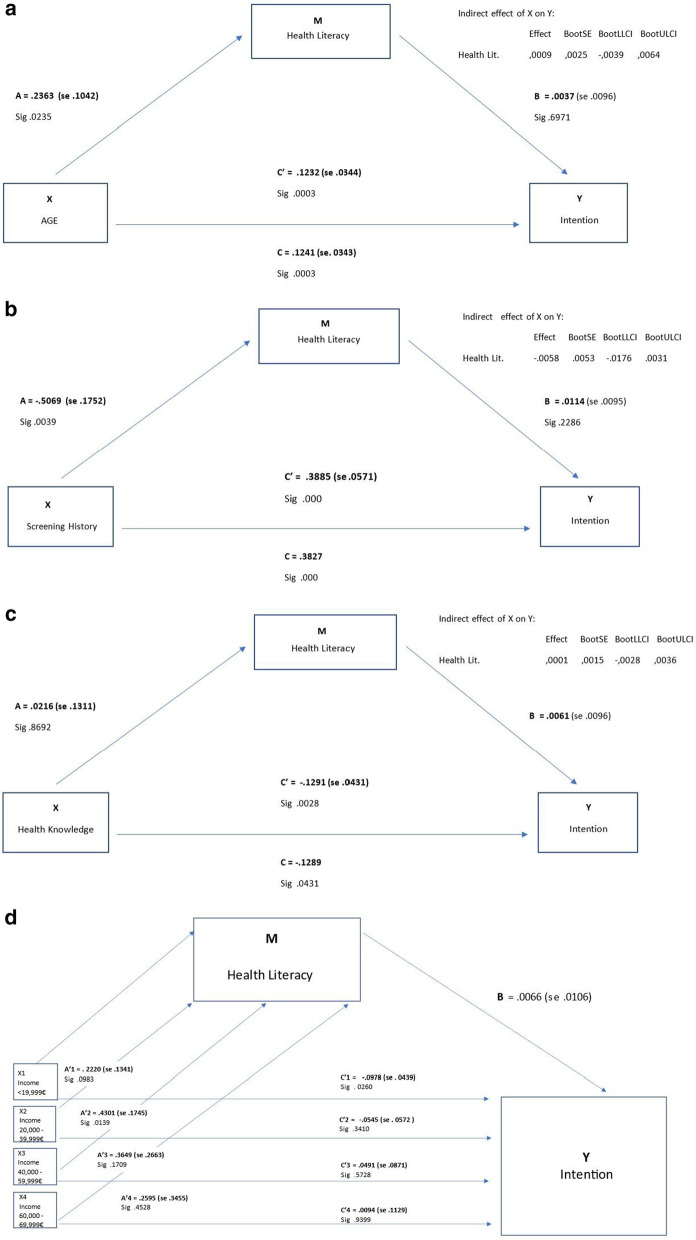
**a** Mediating role of health literacy on relationship between age and intention to be screened amongst women in 5 European countries responding to the survey on mammography screening, 2021. **b** Mediating role of health literacy on relationship between screening history and intention to be screened amongst women in 5 European countries responding to the survey on mammography screening, 2021. **c** Mediating role of health literacy on relationship between health knowledge and intention to be screened amongst women in 5 European countries responding to the survey on mammography screening, 2021. **d** Mediating role of health literacy on relationship between household income and intention to be screened amongst women in 5 European countries responding to the survey on mammography screening, 2021

A mediation analysis was performed to determine the role of health literacy on the relationship between variables corresponding to four individual characteristics addressed in the survey (age, household income, screening history, health knowledge) and intention to screen. Figures 2a, b, c, d show the results from this analysis. The direct relationship between the individual characteristics and intention is significant for all variables with the exception of household income for which only the sub-category of household income < 19.000€ showed a significant association (Fig. 2d). Regarding the association between the variables and health literacy, significant associations were found between age and health literacy (Fig. 2a) and screening history and health literacy (Fig. 2b) but not for health knowledge and health literacy (Fig. 2c). Subsequently partial mediation of health literacy was found in the relationship between the independent variables of age and history of breast cancer screening participation and the dependent variable of intention to screen. However, the effect of health literacy was not statistically significant in each case.

## Discussion

The aim of this study was to investigate if informing women that participation in breast screening has benefits and harms effected their intention to be screened, and to explore the role of several cognitive variables on the outcome. .

Of the nearly 1200 women from five European countries who participated in the survey, only one out of five (19.5%) correctly identified that breast cancer screening carries both benefits and harms, while nearly two out of five (37.9%) responded that breast screening carries no harms at all. This result corresponds to data from systematic reviews which reported that women and health professionals overestimate the benefits of breast screening and underestimate the harms [27, 28]. Being presented with brief information on benefits and harms of breast cancer screening resulted in most participants (56.9%) reporting that they would be more likely to participate in future. This may reflect the high proportion of participants who had previously participated in breast cancer screening (90.3%).

The results suggest that informing about benefits and harms of participation do not necessarily carry a negative effect upon intention to be screened for breast cancer. Prior research has investigated the effect of information on benefits and harms suggesting that, against a baseline of low awareness of the benefits and harms, this information increases knowledge, and informed choice [28], whereas, another study indicated that women becoming better informed about benefits and harms of breast screening may mean they are less likely to choose to participate [29]. Additionally, consistent with the findings of our study, informing about possible risks (such as overdiagnosis) does not affect intention to participate [30–33]. Some studies have indicated that an initial acceptance of harms may decrease over later screening rounds if knowledge about the risks of participation continues to increase from the baseline of low awareness [34, 35]. However, other research indicates that the preferences of women towards information regarding the benefits and harms of breast cancer screening participation are highly heterogenous [36], thus, carefully designed follow up studies would be required to determine such an effect.

Differences by country were observed regarding identifying the presence of harms alongside benefits in breast screening participation, and increased likelihood to be screened in the future. Almost nine out of ten participants from Spain reported that they would be more likely to participate in the future following being informed about the presence of harms alongside benefits of breast screening, which is considerably higher proportion than the result for the total sample. This result may reflect underlying confidence in breast screening by country. A previous study in Spain reported that women have positive views of mammography but were inadequately informed about breast cancer screening, which was interpreted as signifying that women participate on the basis of trust and convenience rather than information [37].

In contrast, only four out of ten of respondents in the United Kingdom and Belgium reported they were more likely to participate in the future. Possible explanations for these results may stem from the age profile of Belgian respondents in which 64.8% of respondents are aged 60 years and older. Consequently, a proportion of the respondents may be beyond the upper age range for breast screening of 69 years old and may, therefore, have no intention of future participation in any case. For the UK, the result may reflect a more established practice to promote balanced information to women eligible for screening target groups, therefore, diminishing the effect of being informed about benefits and harms in this study. This interpretation is supported by result that a greater proportion of UK women (25.9%) than the total sample (19.5%) identified that breast cancer screening carries harms alongside benefits.

For the total sample, many participants (60.7%) could not answer if their chances of developing breast cancer in the next few years were great or not (this corresponded to the cognitive variable of perceived susceptibility). This ranged from 52.5% of respondents in France to 72.4% in Spain, which may indicate that the information provided to women insufficiently communicates about breast cancer risk and may compound inaccuracies in over-estimation of benefit of breast screening. Few respondents (11.1%) agreed with this statement, yet considerable differences by country was observed ranging from 6.7% of women in Italy to 18.9% of women in Spain. Whereas, for women who disagreed with the statement, responses ranged from 8.8% in Spain to 39.1% in France. With this result, the response of Spanish women stand in contrast to the four countries with higher perceived susceptibility of breast cancer suggesting greater need for provision of information to aid informed decision-making.

Examining the effect of the cognitive variables (social norms, perceived barriers, perceived susceptibility, perceived behavioural control), health literacy and background individual characteristics (screening history, health knowledge) on intention to screen, the logistic regression model predicted 20.1% of variance in intention to screen.

A second logistic model was constructed which retained all variables from the initial model (essentially the responses to items of the survey) and incorporated the socio-demographic characteristics of age and household income (proxy of socio-economic status). The predicted variance in the outcome of intention to screen increased marginally with the addition of background individual characteristics of the survey respondents. This suggests that the cognitive variables in the model retained their importance in the relationship with intention. Of the cognitive variables included in the model, perceived behavioural control was the only factor to demonstrate consistently significant results. Social norms also emerged from the analysis as displaying significance in the relationship with intention to be screened as did agreeing with proposition of perceived barriers to screening, which indicated reduced intention to screen. This is consistent with the application of the Theory of Planned Behaviour to predict intention to participate in breast cancer screening, which assumes that perceived behavioural control moderates the effect of social norms on intention [38]. In contrast, health literacy and knowledge of benefits and harms showed limited influence on the outcome of intention. This result suggests that in regards to the information provided to women to make an informed choice about screening, it is important to address perceptions of implementation factors (for example, ease of participation) alongside providing clear and accurate information on benefits and harms.

The limited role of health literacy reported in the logistic regression analysis was also found in the mediation analysis performed to examine the effect of health literacy on background individual characteristics and intention. Considering that the short form of the Health Literacy Survey was used in this study, the analysis may not have been sufficiently sensitivity to explore the impact of health literacy. Additionally, the proportion of respondents who were categorised as demonstrating sufficient health literacy using HLS-EU-6 was noticeably lower than has been reported in previously [39]. This could be attributed to the demographic of the sample population as health literacy correlates negatively with age. Moreover, the fact that more than 90% of the respondents had participated in breast cancer screening previously and that few could identify that breast cancer screening carries harms alongside benefits, could imply that the variables of screening history and knowledge may lack statistical power to detect an effect in this sample. A subgroup analysis of women yet to be screened (due to age) and never screened in their lifetime would be beneficial for future studies.

This study has several strengths and value for practice, owing to its large sample size and the collection of comparable data across several European countries. The descriptive analysis facilitates rapid and informative comparison between countries, indicating underlying trends in awareness of benefits and harms of breast cancer screening. The results also provide data for further investigation into the acceptability of benefits and harms of breast cancer screening across Europe and may inform further research to improve an accurate perception of breast cancer risk amongst women eligible for breast screening. The limited impact of health literacy and the absence of a socio-economic gradient regarding the intention to screen may encourage decision-makers and practitioners to implement straightforward universal information tools and guidance for women.

Despite its strengths, a few important limitations must also be acknowledged with this study. Firstly, whilst a large sample size was included in the study, representativeness to the wider population cannot be guaranteed due to the selection bias inherent to the survey method especially as respondents are active users of a large online research panel. Additionally, the age of the participants was only expressed in a dichotomised age range, prohibiting a more precise analysis on the impact of age and determination of the age profile of the participants. The upper age category of the survey panel is limited to 60 years and older, therefore, we cannot determine if women outside of the typical age range for breast screening in Europe (50–69 years of age) participated.

In addition, the age profile of respondents in each country differed, which may explain some country variations in responses. Regarding socio-demographic characteristics, only household income per annum was used as a proxy of socio-economic status, which could have been further enhanced with the addition of variables such as highest level of education. Finally, the questionnaire items were limited in quantity so that cognitive variables were measured by one item only. This is especially limiting for the measure of knowledge which was narrowed in its scope to being aware that breast screening carries harms alongside benefits. Adding further items per component would have enriched the data and provided greater validity to the effect of each component.

## Conclusions

Our study found that women in five European countries demonstrated low awareness of benefits and harms of breast cancer screening participation in each country. Presentation of brief information about the benefits and harms of participation in breast cancer screening to women did not negatively impact upon their subsequent intention to be screened in the future. The analysis of the influence of factors on intention, ranging from cognitive variables informed by behavioural theories and sociodemographic characteristics, reported a limited role for health literacy. The variables of perceived behavioural control and social norms had significant effect on intention, thus, in regards to the information provided to women to make an informed choice about screening, it is important to address perceptions of implementation factors alongside providing clear and accurate information on benefits and harms. In conclusion, the results from this study suggest that policymakers and programme managers should not be deterred by the assumption of decreased participation through increasing efforts to address the lack of knowledge on benefits and harms in the target population.

## Supplementary Information

**Additional file 1.** Annex: Survey instrument provided to respondents

## Data Availability

Data is available from the authors upon written request.

## Abbreviations

CHBMS: Champion Health Belief Model Scale
CIs: Confidence Intervals
GBCI: Global Breast Cancer Initiative
GBP: Pound Sterling
HBM: Health Belief Model
HLS-EU-Q6: European Health Literacy Survey Questionnaire
ORs: odds ratios
SD: Standard deviation
TPB: Theory of Planned Behaviour
UK: United Kingdom
WHO: World Health Organization
