# Supplementary material for: Factors affecting intention to screen after being informed of benefits and harms of breast cancer screening: a study in 5 European countries in 2021
Source: Arch Public Health. 2022 May 23;80:143. doi: 10.1186/s13690-022-00902-6 (PMC9125943; doi:10.1186/s13690-022-00902-6)
Supplement: Supplementary file 1 — Additional file 1. Annex: Survey instrument provided to respondents [file 13690_2022_902_MOESM1_ESM.pdf]

**ANNEX: SURVEY INSTRUMENT PROVIDED TO RESPONDENTS**

I. Have you ever participated in breast cancer screening (also known as mammography screening)? (**one answer only**):

- ***YES*** *– I was INVITED, by letter for example, by the national/regional breast cancer screening programme*
- ***YES*** *– I was REFERRED by my doctor for a mammogram*
- ***YES*** *– I CANNOT RECALL how I was offered breast cancer screening*
- ***NO*** *– I have NEVER participated in breast cancer screening*

II. Please identify which of the following statements you believe to be correct (**one answer only**):

*Participating in breast cancer screening …*

- *comes with benefits BUT NO harms;*
- *comes with benefits AND harms;*
- *helps prevent cancer BEFORE it occurs.*

*NOTE: order randomised*

III. The correct answer is that participation in **breast cancer screening carries with it some harms** (these are potential risks to the individual) **alongside the benefits** of participation.

**Considering that breast cancer screening comes with benefits AND harms**, which of the following statements best reflects your intention to participate in breast cancer screening in the future **(one answer only):**

- *I am **MORE likely** to participate next time invited/referred for breast cancer screening*
- *I am **LESS likely** to participate next time invited/referred for breast cancer screening*
- *I am **NEITHER more nor less likely** to participate next time invited/referred for breast cancer screening*

*NOTE: order randomised*

## IV. On a scale from very easy to very difficult, how easy would you say it is to …

a. judge when you may need to get a second opinion from another doctor?

b. use information the doctor gives you to make decisions about your illness?

c. find information on how to manage mental health problems like stress or depression?

d. judge if the information on health risks in the media is reliable?

e. find out about activities that are good for your mental well-being?

f. understand information in the media on how to get healthier?

RESPONSES for a. – f.

4-point Likert scales: *1 = very difficult, 2 = difficult, 3 = easy, and 4 = very easy.*

## V. Please answer according to your own personal opinion: Most people who are important to me think I should have my breasts screened ... (**one answer only**):

- *Strongly disagree*
- *Disagree*
- *I am not sure*
- *Agree*
- *Strongly agree*

VI. Please answer according to your own personal opinion: Keeping my appointment for breast cancer screening will be … (**one answer only**):

- *Very difficult*
- *Difficult*
- *I am not sure*
- *Easy*
- *Very easy*

VII. Please answer according to your own personal opinion: My chances of getting breast cancer in the next few years are great ... (**one answer only**):

- *Strongly disagree*
- *Disagree*
- *I am not sure*
- *Agree*
- *Strongly agree*

VIII. Please answer according to your own personal opinion: I have other problems more important than getting a mammogram ... (**one answer only**):

- *Strongly disagree*
- *Disagree*
- *I am not sure*
- *Agree*
- *Strongly agree*
